# The prognostic role of γδ T cells in colorectal cancer based on nomogram

**DOI:** 10.1186/s40001-023-01452-5

**Published:** 2023-10-27

**Authors:** Rulan Ma, Meijun Gong, Tuanhe Sun, Lin Su, Kang Li

**Affiliations:** 1 Department of Surgical Oncology, The First Affiliated Hospital of Xi’an Jiaotong University, 277 West Yanta Road, Xi’an, 710061 Shaanxi China; 2 Biobank, The First Affiliated Hospital of Xi’an Jiaotong University, 277 West Yanta Road, Xi’an, 710061 Shaanxi China

**Keywords:** Colorectal cancer, γδ T cell, Prognosis, Adjuvant chemotherapy, Nomogram

## Abstract

**Objective:**

The aim of the present study was to explore the prognostic role of γδ T cells in colorectal cancer, and establish a nomogram for predicting the survival of the patients.

**Methods:**

Immunohistochemistry was performed to analyze the infiltration degree of γδ T cells in tumor and normal tissues of colorectal cancer. The relationship between γδ T cells infiltration in tumor tissues and the prognosis of patients with colorectal cancer were determined by Cox regression analysis and survival analysis. R software was used to establish and verify a nomogram for predicting the prognosis of patients with colorectal cancer.

**Results:**

The degree of γδ T cell infiltration in tumor tissues and normal tissues of CRC was not different (*t* = 0.35, *P* = 0.73). However, the infiltration of γδ T cell was related to the survival status of the patients (*x*^2^ = 4.88, *P* = 0.03). Besides, the infiltrating degree of γδ T cells in tumor tissue was obviously related to the prognostic improvement of the patients with colorectal cancer (log-rank *P* = 0.02) and could reflect the benefit of adjuvant chemotherapy. The nomogram based on tumor diameter, tumor location, AJCC stage, chemotherapy, serum CEA level and γδ T cell infiltration was established and could provide a reference for predicting the survival of colorectal cancer patients.

**Conclusion:**

γδ T cell infiltration degree in tumor tissue was an important factor to improve the outcome of patients with colorectal cancer, and can predict the benefit of adjuvant chemotherapy.

## Introduction

Colorectal cancer (CRC) is one of the most malignant tumors. Globally, the incidence of CRC ranks 3rd, and CRC is the 2nd leading cause of tumor death [1]. According to the latest research, about 1.8 million patients worldwide were diagnosed with CRC, and about 0.86 million patients died of CRC in 2018 [2]. Although the mortality rate of CRC has shown an overall descending trend in the last several decades, the incidence of CRC has increased year by year among patients under the age of 50 [3, 4]. At present, surgery-based treatment is mainly aimed at CRC patients in the early stages. However, for patients with advanced CRC, especially those with distant metastasis, radical surgery cannot be performed, and the progress of CRC can only be controlled by radiotherapy, chemotherapy, endocrine therapy or immunotherapy. Hence, it is crucial to search for a novel prognostic factor and treatment target for CRC.

γδ T cells are a unique subset of T lymphocytes and an important component of the innate immune system in the human body, participating in the formation of the first line of defense against pathogens [5]. They can recognize and kill tumor cells in a manner that does not depend on MHC-I molecules. Researches have shown that γδ T cells can directly kill tumor cells by secreting cytokines such as IFN-γ, TNF-α, perforin, granzyme B, and they can also exert anti-tumoral effect through the death receptor-ligand pathway, antibody-mediated cell cytotoxicity, or direct contact [6–9]. However, some studies have also shown that γδ T cells can exert a pro-tumoral effect by recruiting myeloid-derived suppressor cells, inflammatory monocytes, and neutrophils through secreting IL-17 and other pro-inflammatory cytokines [10–14]. Previous studies on the prognosis of γδ T cells in tumors have shown that in gastric cancer, the degree of infiltration of γδ T cells is related to improved patient prognosis and benefits from postoperative adjuvant chemotherapy [15]. However, in other tumors such as breast cancer, gallbladder cancer, and ovarian cancer, the increased infiltration of γδ T cells in tumor tissue is closely related to poor prognosis [16–18]. These suggest that γδ T cells play different roles in different types of tumors.

In CRC, Meraviglia S and Gentles AJ et al. reported that increase of γδ T cells could obviously improve the prognosis of patients [7, 19]. However, these two studies are bioinformatics analysis and lack experimental verification. A later study suggested that the sorting algorithm used by Gentles AJ et al. could not accurately distinguish CD8^+^ T cells, γδ T cells and NK cells because of the overlap of transcriptome [20]. Therefore, the elevated infiltration of γδ T cells was not always related to the better prognosis of CRC patients [20]. Besides, Pin Wu et al. confirmed the positive correlation between the degree of infiltration of γδT17 cells in tumor tissues and the clinical and pathological characteristics of CRC patients, as well as its promoting effect on human CRC and its mechanism [14]. These studies indicate that the role played by γδ T cells in the prognosis of CRC is still controversial. Therefore, it is necessary to further validate the relationship between γδT cell infiltration and the prognosis and clinical pathological characteristics of CRC cancer patients.

Thus, in this study, we aimed to confirm the relationship of γδ T cell infiltration with the prognosis of CRC patients and establish a prediction model for forecasting survival rate of CRC patients based on γδ T cell infiltration degree, thereby providing a new prognostic indicator for CRC patients.

## Methods

### Patients

Fifty-eight paraffin-embedded tumor tissue samples and 20 matched adjacent normal tissue samples were collected. All CRC patients received operations from January 2010 to December 2013 in the First Affiliated Hospital of Xi’an Jiaotong University. The inclusion criteria were as follows: (1) age: 18–75 years old; (2) postoperative pathology: CRC; (3) the clinicopathological data and follow-up data were completed. This study was carried out under the approval and supervision of the Ethics Committee of The First Affiliated Hospital of Xi’an Jiaotong University (No. XJTU1AF2022LSK-250).

### Immunohistochemistry

Tissue specimens were embedded in paraffin, sliced into serial 4-mm sections. The slides were deparaffinized, rehydrated and incubated with the primary antibody (anti-TCR gamma/delta antibody, VA288448, 1:100 dilution, Invitrogen, USA) overnight at 4 °C. Then, the slides were incubated with the secondary antibody (Goat anti-rabbit IgG, 1:200 dilution, Thermo Fisher Scientific, USA) for 60 min at 24 °C. The slides were stained with hematoxylin, dehydrated and slip-covered. Images of stained slides were obtained using an optical microscope.

The sections were observed under a microscope at low magnification. Yellow or brown cell were recognized as the positive cells. The Leica Microsystems was used to scan the slice at 200 × magnification. Qualitative and quantitative analysis was performed by using IHC Profiler plug-in of Image J software. The bar value was set to 100 μm. Five visual fields were randomly selected for each slice to determine the positive rate, and the average value was recognized as the positive expression rate of γδ T cells in this slide. The average positive rate of γδ T cells in per three consecutive sections was taken as the degree of infiltration of γδ T cells in the tumor or adjacent normal tissues of the patient. Our study showed that the cut-off value of γδ T cell infiltration in tumor tissue was 57.32%, thus defining ≥ 57.32% as high γδ T cell infiltration and < 57.32% as low γδ T cell infiltration.

### Statistical analysis

The statistical analysis was performed by SPSS 26.0 and Graphpad Prism 8.0. The data are expressed as x ± S or ratio. The differences between the two groups are analyzed by t-test, Chi-squared test or Fisher’s exact probability method. Univariable and multivariable analysis was conducted by using Cox regression analysis. The factors with *P*-value < 0.05 in univariable analysis into further multivariable analysis. Kaplan-Meier method was used to analyze the cumulative survival rate, and the log-rank test was used to analyze the survival difference. According to the result of multivariable Cox regression analysis, a nomogram was established by using R 3.6.2. 1000 Bootstrap resampling internal verification and calibration curve were performed to validate the nomogram. The accuracy of the nomogram was evaluated by concordance index (C-index). The closer the C-index is to 1, the more accurate the prediction ability of the nomogram.

## Results

### The clinicopathological features of the patients

In our study, the tumor tissues of 58 CRC patients and 20 matched adjacent normal tissues were included. Among these patients, 33 cases are males and 25 cases are females, with an average age of 60.45 ± 2.18 years old. The basic clinical and pathological features of the patients are shown in Table 1.

**Table 1:** Clinicopathological features of 58 patients with colorectal cancer

Variables	Items	N	%
Gender	Male	33	56.90
	Female	25	43.10
Age (Y)	≥ 50	50	86.21
(60.45 ± 12.18)	< 50	8	13.79
Tumor location	Rectum	40	68.97
	Right semi-colon	10	17.24
	Left semi-colon	5	8.62
	Whole colon	3	5.17
Tumor diameter (cm)	≥ 5	25	43.10
(4.25 ± 1.47)	< 5	33	56.90
Grade	I	3	8.57
	II	29	82.86
	III	3	8.57
T	Tis	1	1.72
	1	2	3.45
	2	11	18.97
	3	31	53.45
	4	13	22.41
N	0	38	65.52
	1	14	24.14
	2	6	10.34
M	0	51	87.93
	1	7	12.07
AJCC stage	0	1	1.72
	1	12	20.69
	2	23	39.66
	3	15	25.86
	4	7	12.07
Intravascular infiltration	Yes	1	1.72
	No	57	98.28
Chemotherapy	Yes	26	44.83
	No	32	55.17
Radiotherapy	Yes	54	93.10
	No	4	6.90
CEA (ng/mL)	≥ 5	18	31.03
(28.38 ± 88.40)	< 5	40	68.97

### Infiltrating degree of γδ T cells in tumor tissues and adjacent normal tissues of CRC patients

**Fig. 1:**
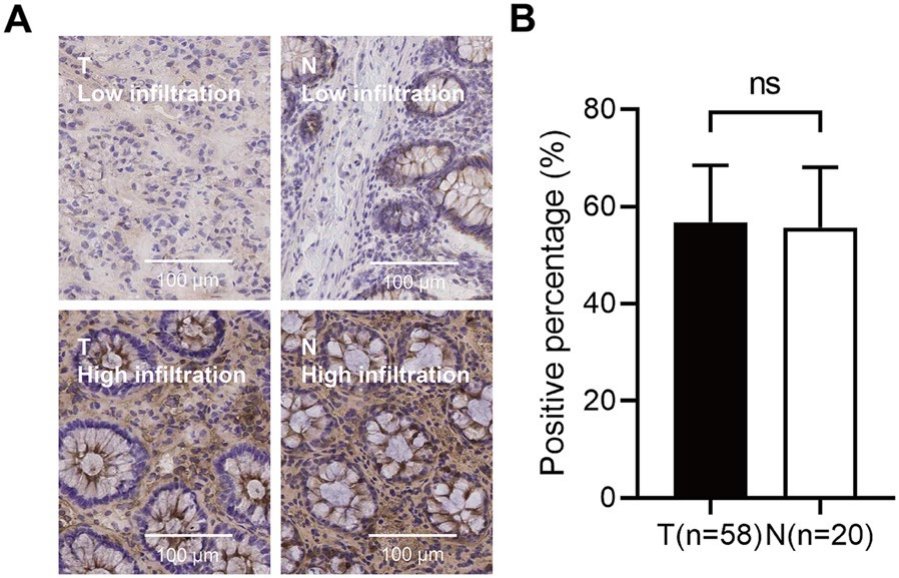
Expression of γδ T cells in tumor tissues and adjacent normal tissues in colorectal cancer. **A** Results of immunohistochemistry. **B** Quantitative analysis. *T* tumor tissues, *N* adjacent normal tissues

The result of immunohistochemistry showed that the positive rate of γδ T cell expression in tumor tissues was 56.72% ± 11.72%, while that in adjacent normal tissues was 55.66% ± 12.39% (Fig. 1A, B). Student’s t-test showed that the relative abundance of γδ T cells in CRC tumor tissues was not different from the infiltrating degree of γδ T cells in adjacent normal tissues (t = 0.35, *P* > 0.05) (Fig. 1B).

### The relationship between the tumor-infiltrating degree of γδ T cells and the clinicopathological features of patients with CRC

The relationship between clinicopathological characteristics and the degree of γδ T cell infiltration in tumor tissue was analyzed by x^2^ test. The results suggested that the infiltrating degree of γδ T cells in tumor tissue was not related to age, gender, tumor size, TNM stage and so on, but it was relevant to the survival status of the patients (x^2^ = 4.884, *P* = 0.027) (Table 2).

**Table 2:** The relationship between γδ T cell infiltration and the clinicopathological features of patients with colorectal cancer

Variables	Items	γδ T infiltration		*P*-value
		High	Low	
Gender	Male	13	12	0.79
	Female	16	17	
Age (Y)	≥ 50	4	4	1.00^a^
	< 50	25	25	
Tumor location	Rectum	19	21	0.57
	Colon	10	8	
Tumor diameter (cm)	≥ 5	12	13	0.79
	< 5	17	16	
Grade	I	2	1	0.60^a^
	II/III	15	17	
T	Tis/0/1/2	6	8	0.54
	3/4	23	21	
N	0	17	21	0.27
	1/2	12	8	
M	0	23	28	0.10^a^
	1	6	1	
AJCC stage (Version 8.0)	0/I/II	16	20	0.28
	III/IV	13	9	
Adjuvant chemotherapy	Yes	17	15	0.60
	No	12	14	
Adjuvant radiotherapy	Yes	3	1	0.61^a^
	No	26	28	
CEA (ng/mL)	≥ 5	11	7	0.26
	< 5	18	22	
Survival status	Survive	23	15	**0.03**
	Death	6	14	

^a^ Using Fisher’s exact probability method to analyze the difference between two groups

### The relationship of γδ T cell infiltration degree in tumor tissues with the prognosis of patients with CRC

**Fig. 2:**
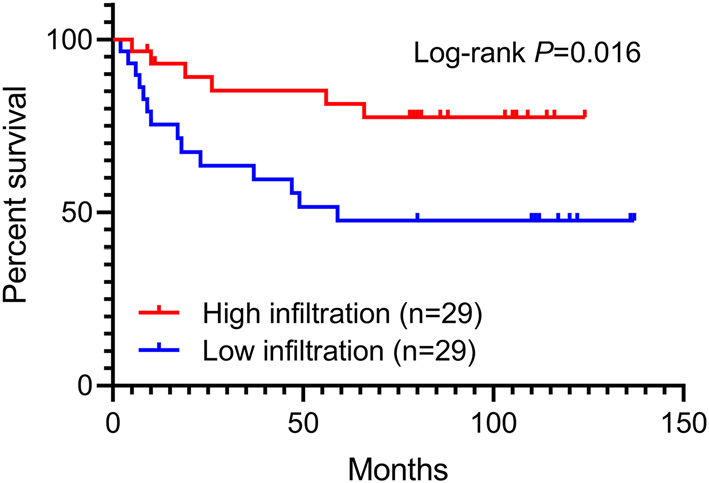
The relationship of the infiltration of γδ T cells in tumor tissues with the prognosis of patients with colorectal cancer

In order to determine whether the tumor-infiltrating degree of γδ T cells was related to the prognosis of the patients with CRC, we utilized the Kaplan-Meier method to calculate the cumulative survival rate of patients with low or high γδ T cell infiltration. The difference in the survival rates between the two groups was evaluated by the log-rank test. The results showed that the lower tumor-infiltrating degree of γδ T cells was related to the poorer prognosis of CRC patients, while the higher the infiltrating degree of γδ T cells in tumor tissues, the better the prognosis (log-rank *P* = 0.016) (Fig. 2).

### Factors associated with the prognosis of CRC patients by Cox regression analysis

**Fig. 3:**
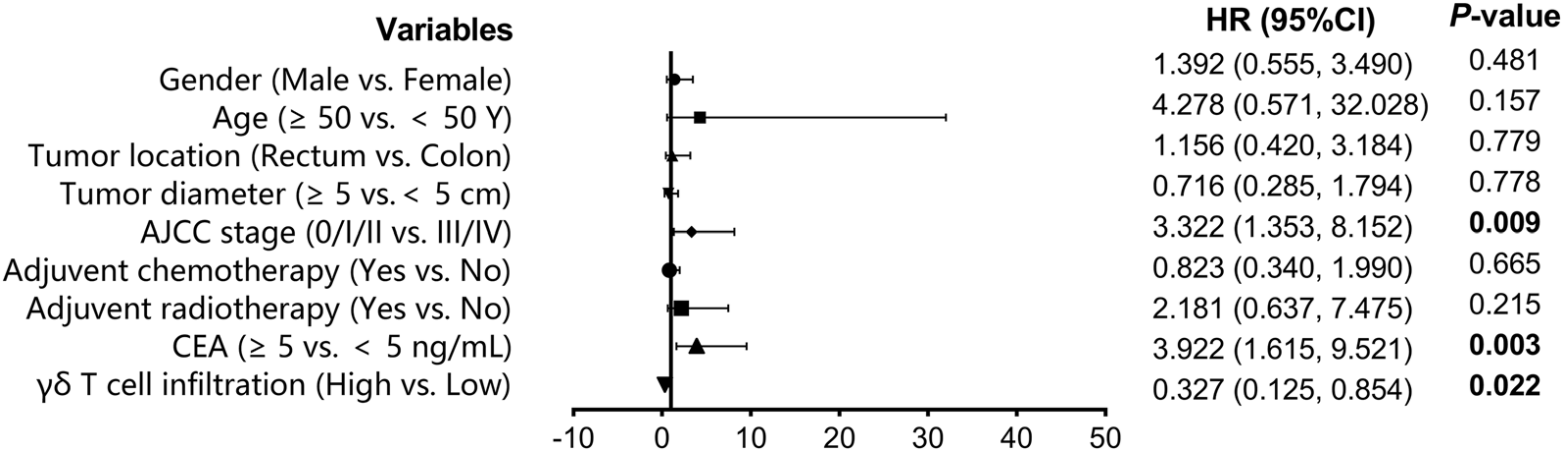
Univariable Cox regression analysis of prognostic factors of colorectal cancer

**Fig. 4:**
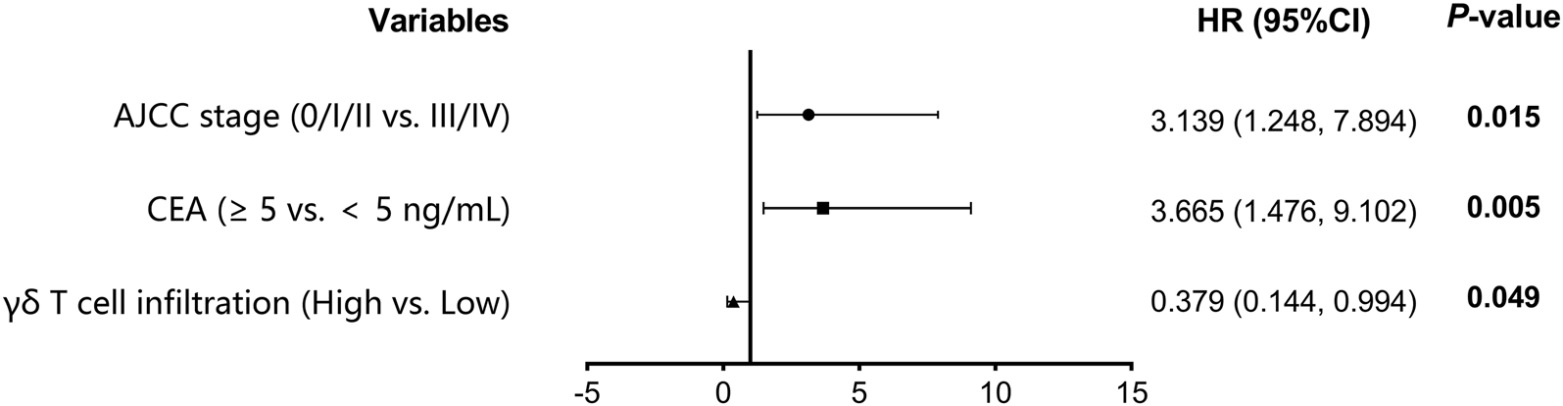
Multivariable Cox regression analysis of prognostic factors of colorectal cancer

We used Cox regression analysis to explore the factors that might impact the overall survival of the patients with CRC. The result of the univariable analysis suggested that AJCC stage (HR:3.322, 95%CI 1.353–8.152, *P* = 0.009), serum CEA level (HR:3.922, 95%CI 1.615–9.521, *P* = 0.003) and the abundance of tumor-infiltrating γδ T cells (HR:0.327, 95%CI 0.125–0.854, *P* = 0.022) were the factors related to the prognosis of CRC patients (Fig. 3). Further multivariable analysis indicated that AJCC stage (HR 3.139, 95%CI 1.248–7.894, *P* = 0.015), serum CEA level (HR:3.665, 95%CI 1.476–9.102, *P* = 0.005) and γδ T cell infiltration degree in tumor tissue (HR:1.379, 95%CI:0.144,0.994, *P* = 0.049) were the independent prognostic factors correlated with the prognosis of patients with CRC (Fig. 4).

### Infiltrating degree of γδ T cells was related to the survival benefit of adjuvant chemotherapy for CRC patients

**Fig. 5:**
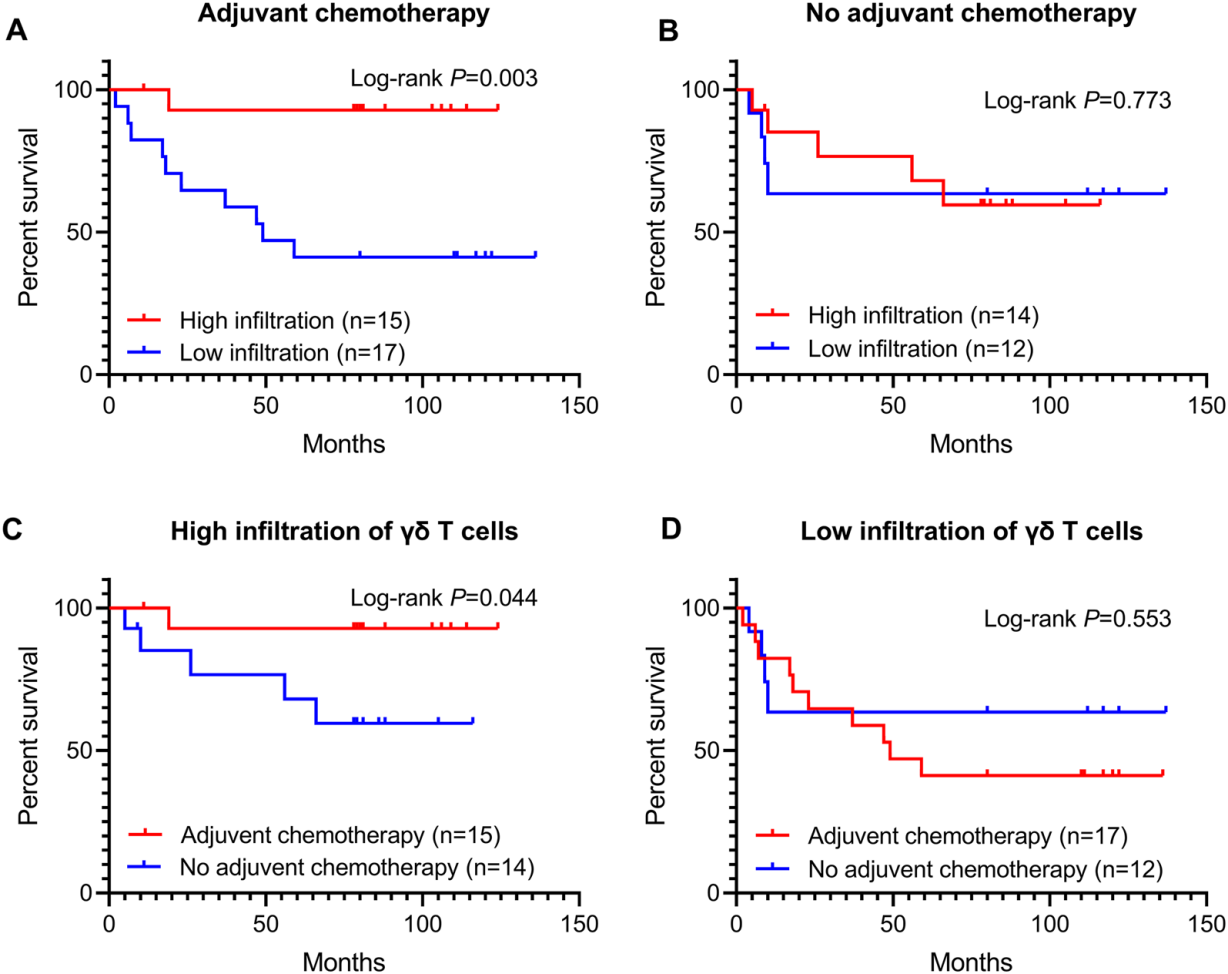
The relationship between γδ T cell infiltration and the survival benefit of postoperative adjuvant chemotherapy. **A** Patients with adjuvant chemotherapy. **B** Patients without adjuvant chemotherapy. **C** Patients with high γδ T cells infiltration. **D** Patients with low γδ T cells infiltration

Given that γδ T cell infiltration in tumor tissue is a critical factor related to the prognosis of CRC patients, we further analyzed the relationship between the infiltrating degree of γδ T cells and the prognosis of patients who received adjuvant chemotherapy. We found that in patients who received postoperative adjuvant chemotherapy, high γδ T cell infiltration was associated with the improved prognosis of CRC patients (log-rank *P* = 0.003) (Fig. 5A, B). Besides, in patients with high infiltration of γδ T cells, adjuvant chemotherapy could obviously improve the prognosis of CRC patients (log-rank *P* = 0.444) (Fig. 5C, D). Taken together, the degree of γδ T cell infiltration in CRC tumor tissues can predict whether patients can benefit from postoperative adjuvant chemotherapy.

### Establishment and validation of the nomogram for predicting the prognosis of CRC patients

**Fig. 6:**
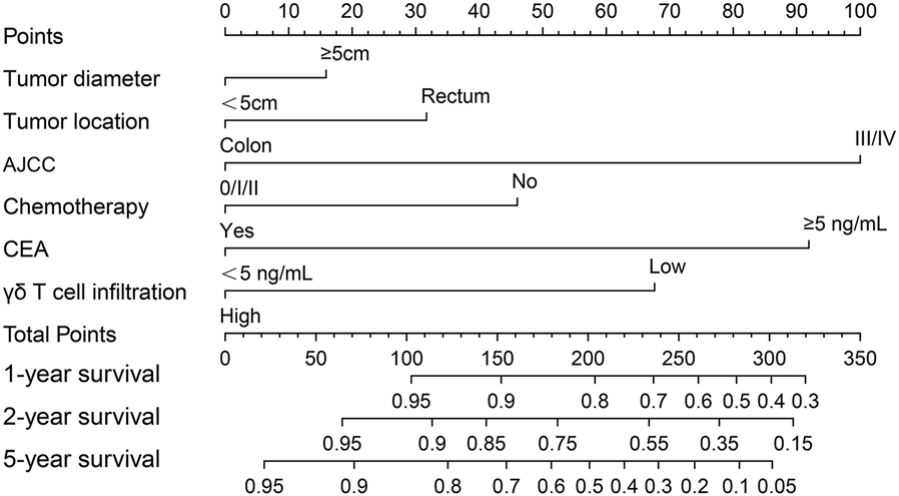
The nomogram for predicting the prognosis of patients with colorectal cancer

**Fig. 7:**
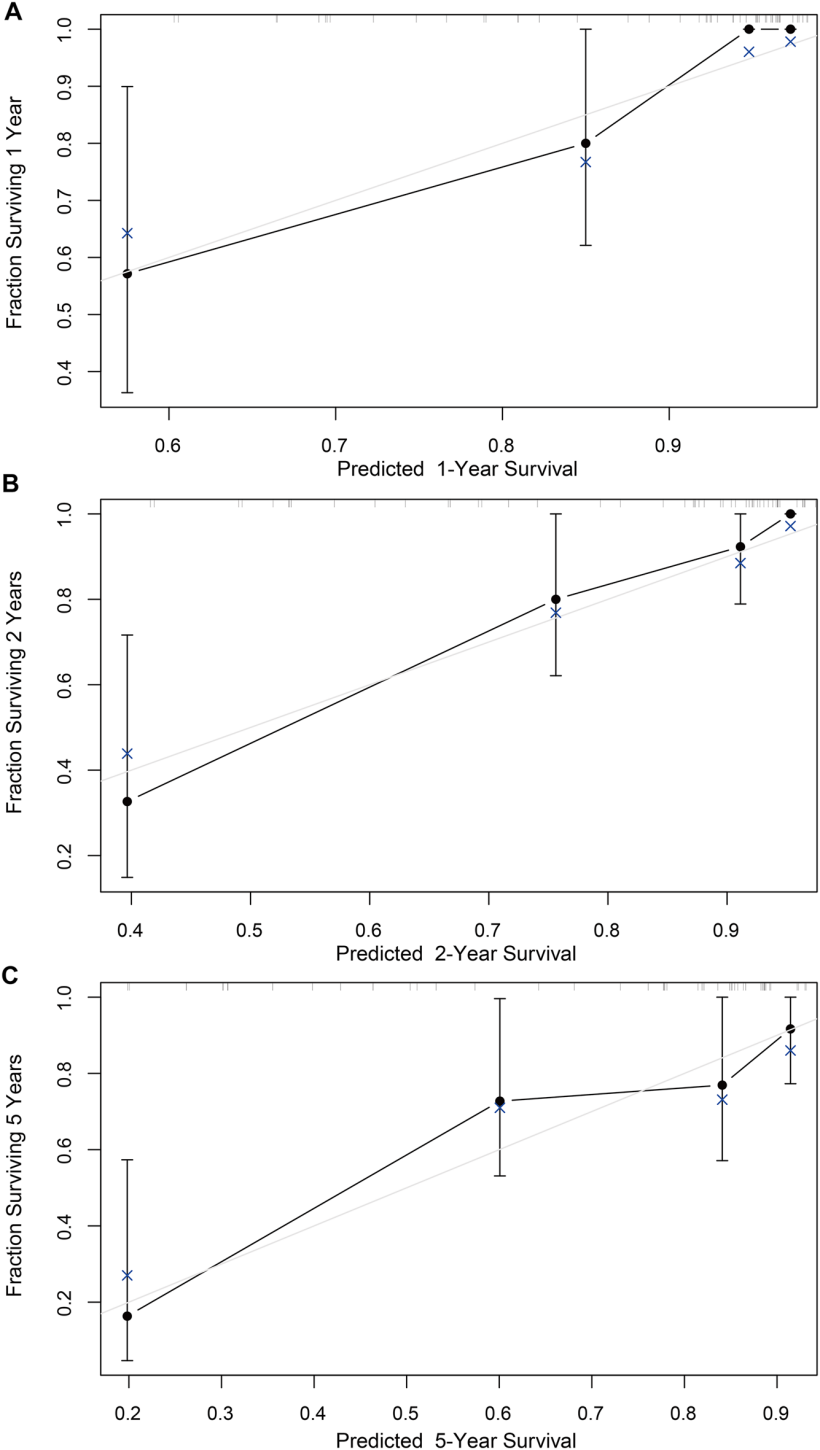
The calibration curves for 1-, 2-, 5-year survival. **A** The calibration curve for 1-year survival. **B** The calibration curve for 2-year survival. **C** The calibration curve for 5-year survival

According to the multivariate Cox regression analysis, we included AJCC stage, serum CEA level and γδ T cell infiltration degree into the establishment of the nomogram. Given that tumor diameter [21], tumor location [22] and chemotherapy [23] could impact the prognosis of the patients, we also included these three factors into the development of the nomogram. The nomogram aimed to predict the prognosis of CRC patients was developed as shown in Fig. 6. The predicted rate of 1-, 2-, 5-year survival can be obtained by summing the scores of the six factors. The C-index was 0.788 (95% confidence interval: 0.695–0.881), indicating the discrimination of the nomogram is relatively good. The calibration curves for 1-, 2-, 5-year survival also showed the relative accuracy of nomogram in predicting the prognosis of patients (Fig. 7A–C).

## Discussion

As the first line for the anti-infection and anti-tumor immune response, it was proved that γδ T cells could infiltrate into tumor tissue and exert an essential role. Previous studies have shown that γδ T cells play different roles in different types of tumors. For example, increased infiltration of γδ T cells in tumor tissues is an independent protective factor for the prognosis of gastric cancer patients [15]. However, in tumors such as breast cancer, pancreatic cancer, ovarian cancer, and gallbladder cancer, the degree of γδ T cell infiltration is positively correlated with poor prognosis in patients [11, 16–18, 24]. However, in CRC, the relationship between γδ T cell infiltration and patient prognosis is still controversial [7, 14, 19, 20]. Therefore, in this study, we further clarified the relationship between the degree of γδ T cell infiltration and the prognosis of CRC patients through immunohistochemical staining and prognosis analysis.

Through immunohistochemical analysis, we found that although there was no significant difference in the relative abundance of γδ T cells between tumor tissue and adjacent normal tissue, the abundance of γδ T cells in tumor tissue was significantly correlated with the survival status of patients with CRC. The results of survival analysis also indicated that the increased γδ T cell infiltration could improve the survival rate. Patients with a high level of γδ T cell infiltration acquired a better prognosis after receiving postoperative chemotherapy. Meanwhile, among patients who received adjuvant chemotherapy, the cumulative survival of patients with the increased γδ T cell infiltration was better than that of patients with lower γδ T cell infiltration. These results suggested that the tumor-infiltrating γδ T cells, regardless of subtypes, was positively correlated with the improvement of the prognosis of CRC patients.

A previous study showed the expression of γδ T cells in tumor and adjacent tissues was not different in rectal cancer [25], which was consistent with the result of our study. Similarly, S. Meraviglia et al. found that the abundance of γδ T cells in tumor tissues was not significantly different from that in non-tumor colon tissues by analyzing the abundance and composition of γδ T cell subtypes (Vδ1 T cells and Vδ2 T cells) in CRC [7]. However, their study showed that most of the γδ T cells in tumor tissues and adjacent normal tissues are Vδ 1 T cell subtypes and showed effector phenotypes. Moreover, their ability to produce IL-17 was insufficient, which was contrary to the conclusion that γδ T cells were the main producers of IL-17 in CRC studied by Pin Wu et al. [14]. Their further analysis found that this might be caused by some inhibitory molecules produced by tumor stem cells in the tumor microenvironment [7]. Of note, S. Meraviglia et al. also found that there was no significant difference in the abundance of γδ T cells in tumor tissues and normal tissues [7]. But their bioinformatic analysis of transcriptome data indicated that CRC patients with enrichment of γδ T cells in tumor tissues had a longer 5-year disease-free survival [7], which was consistent with our results. In addition, our study suggested that the abundance of γδ T cells was related to whether patients could benefit from postoperative adjuvant chemotherapy, which was consistent with previous studies in gastric cancer [15]. The reason why our results were not consistent with those of other researchers might be mainly related to the difference in the effects of γδ T cells and other factors in the different tumor microenvironments. How the tumor-infiltrating γδ T cells play an inhibitory role in CRC is still a problem that we need to further explore and prove.

The nomogram is a prediction model based on multivariate regression analysis [26]. It can predict the probability of a specific event and present the relationship of the prediction model with the predicted factors. According to the result of the Cox regression analysis, we established a nomogram based on tumor diameter, tumor location, AJCC stage, chemotherapy, serum CEA level and γδ T cell infiltration. The C-index and the calibration curve suggested that the nomogram had a relatively good ability for predicting survival of patients with CRC. This nomogram could provide a reference for predicting the 1-, 2-, 5-year survival rate of CRC patients in clinical practice.

However, the limitations in this study cannot be ignored. First, the sample size in this study is small. Although we obtained some positive results, the verification based on large sample is still necessary. Second, only immunohistochemistry analysis is used for detecting the infiltration of γδ T cells in CRC. And the relationship between subtype of γδ T cells and the prognosis of the patients is not evaluated. Next, we need to verify these by qPCR, flow cytometry and so on.

Nonetheless, we can conclude that the infiltration of γδ T cells in tumor tissue is an important protective factor for the prognosis of patients with CRC, and the abundance of tumor-infiltrating γδ T cells can predict the benefit of patients from postoperative adjuvant chemotherapy.

## Data Availability

The datasets used and analyzed during the current study are available from the corresponding author on reasonable request.
